# Immunomodulatory role for membrane vesicles released by THP-1 macrophages and respiratory pathogens during macrophage infection

**DOI:** 10.1186/s12866-017-1122-3

**Published:** 2017-11-13

**Authors:** Charlotte Volgers, Birke J. Benedikter, Gert E. Grauls, Paul H. M. Savelkoul, Frank R. M. Stassen

**Affiliations:** 1 Department of Medical Microbiology, School of Nutrition and Translational Research in Metabolism (NUTRIM), Maastricht University Medical Centre, P. Debyelaan 25, 6229 HZ Maastricht, The Netherlands; 2 Department of Medical Microbiology and Infection Control, VU University medical center, Amsterdam, The Netherlands

**Keywords:** Membrane vesicles, Extracellular vesicles, Outer membrane vesicles, Bacterial infection, Inflammatory response, Immuno-modulation, Non-typeable *Haemophilus influenzae*, *Moraxella catarrhalis*, *Streptococcus Pneumoniae*, *Pseudomonas Aeruginosa*

## Abstract

**Background:**

During infection, inflammation is partially driven by the release of mediators which facilitate intercellular communication. Amongst these mediators are small membrane vesicles (MVs) that can be released by both host cells and Gram-negative and -positive bacteria. Bacterial membrane vesicles are known to exert immuno-modulatory and -stimulatory actions. Moreover, it has been proposed that host cell-derived vesicles, released during infection, also have immunostimulatory properties. In this study, we assessed the release and activity of host cell-derived and bacterial MVs during the first hours following infection of THP-1 macrophages with the common respiratory pathogens non-typeable *Haemophilus influenzae, Moraxella catarrhalis, Streptococcus pneumoniae,* and *Pseudomonas aeruginosa*.

**Results:**

Using a combination of flow cytometry, tunable resistive pulse sensing (TRPS)-based analysis and electron microscopy, we demonstrated that the release of MVs occurs by both host cells and bacteria during infection. MVs released during infection and bacterial culture were found to induce a strong pro-inflammatory response by naive THP-1 macrophages. Yet, these MVs were also found to induce tolerance of host cells to secondary immunogenic stimuli and to enhance bacterial adherence and the number of intracellular bacteria.

**Conclusions:**

Bacterial MVs may play a dual role during infection, as they can both trigger and dampen immune responses thereby contributing to immune defence and bacterial survival.

## Background

Inflammation in response to bacterial infection results from contact of host cells with bacteria or bacterial products and this process is directed by an extensive communication between cells of the infected tissues, immune cells, and bacteria [1, 2]. Involved in this communication are small soluble mediators as well as nanosized membrane vesicles (MVs) that are released by bacteria and by host cells [3–6].

Bacterial MVs have gained a considerable interest over the past decades as they are involved in physiological and pathological processes by transferring functional vesicular cargos to recipient cells or by direct vesicle-mediated target cell activation in bacteria–host cell interactions. The release of MVs by Gram-negative bacteria has been studied extensively (as reviewed by Schwechheimer and Kuehn [4, 7]). More recently it has been revealed that Gram-positive bacteria also shed MVs (as reviewed by Brown et al. [8]). MV release takes place not only during bacterial culture but also during infection, both in vitro and in vivo [7–9]. Yet, only a few studies assessed bacterial MV shedding during infection, and consequently the kinetics for release as well as the functionality of MVs shed during infection have remained poorly addressed [10–12].

From the perspective of the host, previous studies have shown that MVs shed upon infection with intracellular pathogens have immunostimulatory and -modulatory properties [13–15]. Initially it was anticipated that this was the result of the incorporation of bacterial components into host MVs. However, this has been questioned recently by findings of Athman et al. showing that bacterial cargo-containing MVs, shed upon infection with *Mycobacterium tuberculosis,* are mostly of bacterial origin [13]. Thus, MVs released during infection are most likely a complex mixture of both bacterial and host-derived MVs.

To obtain clarity on the dynamics of MV shedding during infection, we studied the release kinetics and examined the functional activity of both host cell-derived and bacterial MVs released by THP-1 macrophages infected with non-typeable *Haemophilus influenzae* (NTHi)*, Moraxella catarrhalis* (Mrc)*, Streptococcus pneumoniae* (Spn), and *Pseudomonas aeruginosa* (Psa), which are well-known opportunistic pathogens most frequently involved in lower respiratory tract infections in humans [14–16]. We found that MV release occurs by both the host and bacteria during infection, by bacteria during culture and that both MV populations have pro-inflammatory and immuno-modulating properties.

## Methods

### Cells and media

Human monocytic cells (THP-1, American Type Culture Collection (ATCC)-TIB202, Manassas, VA, USA) were cultured at 5% CO_2_ and 37 °C in RPMI1640 (Sigma, St. Louis, MO, USA) containing 10% fetal calf serum (FCS) (Lonza, Verviers, Belgium) with glucose (0.045%), sodium pyruvate (200 μM), and β-mercaptoethanol (50 μM). For monocyte differentiation, cells were stimulated for 72 h with 200 nM phorbol 12-myristate 13-acetate (PMA; Sigma, St. Louis, MO, USA) at 1 × 10^7^ cells per flask in T75-flasks, at 0.5 × 10^6^ cells per well when using a 24-well plate, and at 1 × 10^4^ per well for 96-well plates. After differentiation, the medium was replaced with complete culture medium with 5% FCS which had previously been depleted of vesicles. Vesicle-depleted medium was obtained by the overnight centrifugation of RPMI1640 containing 30% FCS, glucose, and sodium pyruvate at 100.000×*g* using a 70Ti-rotor, κ-factor 44 in an Optima L-90 K ultracentrifuge (both Beckman Coulter, Fullerton, CA, USA). By combining this medium with FCS-free RPMI1640 medium with glucose and sodium pyruvate, 5% FCS vesicle-free culture medium was obtained.

### Reagents and antibodies

Antibodies against CD63 (unconjugated, mouse-anti-human clone H5C6) and CD81 (PE conjugated, mouse-anti-human clone JS-81) were obtained from BD (BD Biosciences, Franklin Lakes, NJ, USA). Purified lipopolysaccharide (LPS) from *Escherichia coli* was purchased from Sigma (St. Louis, MO, USA). Pam3CSK4 was obtained from InvivoGen (InvivoGen, San Diego, CA, USA). Polymyxin B sulfate salt was from Sigma (St. Louis, MO, USA).

### Bacterial strains, culture conditions, and heat-inactivation

The following bacterial strains were used: NTHi (ATCC-49247), Psa (ATCC-27853), Spn (ATCC-49619), and a clinical Mrc isolate obtained at the academic hospital of Maastricht (Academic Hospital Maastricht (AZM), the Netherlands). All strains were pre-cultured overnight on blood plates at 5% CO_2_ and 37 °C, except for NTHi which was pre-cultured on VitaleX-supplemented chocolate agar plates (Oxoid, Wesel, Germany). After overnight growth, several colonies were picked and resuspended at 0.5 McFarland (1.5 × 10^8^ colony forming units (cfu) ml^-1^) in RPMI1640. These suspensions were then used for infection or culture experiments. For bacterial culture, bacteria were added to 30 ml of vesicle-depleted culture medium supplemented with 5% FCS, prepared as described in the cells and media section, and cultured for 4 h. For heat-inactivation, bacterial suspensions were exposed for 60 min to 65 °C. After heat-inactivation, the inactivated bacteria were washed twice with phosphate buffered saline (PBS) and resuspended at 0.5 McFarland. Inactivation was confirmed by overnight plating of 150 μl of the bacterial suspension on blood plates at 5% CO_2_ and 37 °C, except for NTHi which was cultured on vitalex-supplemented chocolate agar plates.

### Infection or stimulation of THP-1 macrophages with (heat-inactivated) bacteria

Infection and stimulation of THP-1 macrophages was performed at an MOI of 10. For vesicle isolation infections were performed in T75-flasks. 1 × 10^7^ adherent THP-1 macrophages were washed twice with PBS after which they were infected, stimulated with heat-inactivated bacteria, or left untreated in 30 ml of vesicle-depleted culture medium for 4 h. Next, the culture medium was harvested for MV isolation. For semi-quantitative MV analysis by bead-based flow cytometry, cells were infected or stimulated for 2–8 h on 24-well plates and the supernatants were collected.

### Determination of the number of intracellular bacteria

THP-1 macrophages seeded on 24-well plates were infected for 2–8 h. Hereafter the cells were washed 3 times with PBS, then medium containing 300 μg/ml gentamicin was added and after 2 h, cells were washed 3 times with PBS, lysed by adding 300 μl distilled water with 0.025% saponin for 10 min, and neutralized using 700 μl culture medium. The number of adherent and intracellular bacteria was then determined by bacterial plating of 2 different dilutions.

### Cytotoxicity assay

A dimethylthiazol diphenyltetrazolium bromide (MTT) assay was used to determine the cell viability. THP-1 macrophages seeded in 24-well plate were infected for 2–8 h, washed with PBS, whereafter medium containing 0.5 mg/ml MTT (Sigma, St. Louis, MO, USA) was added. Cells were incubated for 1 h, then the medium was removed and DMSO was added to dissolve the formazan crystals. The optical density (OD) at the wavelength of 540 nm was determined and the OD relative to uninfected control cells was used to determine the percentage of viable cells.

### MV concentration and purification from conditioned medium using ultrafiltration and size exclusion chromatography (SEC)

Conditioned media (30 ml) obtained upon infection or culture were centrifuged at 300×*g* for 10 min followed by two 1200×*g* centrifugation steps for 10 min. After this the supernatants were filtered through 0.22-μm filters. Hereafter 2 ml samples were taken and stored at -80 °C until further analysis. The remaining supernatants were concentrated by ultrafiltration (as described by Lobb et al. [17]) and purified by SEC. For ultrafiltration, the conditioned media were concentrated to 500 μl in 2 runs at 4000×*g* for 15 min at room temperature using Amicon ultra-15 10-kDa centrifugal filter units (Millipore, Billerica, MA, USA). The concentrates were then purified by sepharose columns as described by Boing et al. with some minor modifications [18]. Briefly, 30 ml sepharose CL-2B (GE Healthcare, Uppsala, Sweden) was washed twice with PBS, and 10 ml sepharose was used to stack TELOS 15 ml filtration column (Kinesis Scientific Experts, St. Neots, Cambridgeshire, UK). Next, the concentrates were loaded onto the column and fractions of 0.5 ml were eluted using PBS. Fractions 7–11 were found to be highly enriched for MVs and negative for free protein (determined by flow cytometry and microBCA). These fractions were pooled and stored at -80 °C until use. For electron microscopic analysis, the isolations were performed according to the same protocol except for the initial bacterial culture or cell culture conditions: bacterial culturing was performed overnight using a total 2 × 10^9^ cfu in a total of 60 ml RPMI without FCS. Conditioned medium from THP-1 macrophages was obtained from 4 T75-flasks containing 1 × 10^7^ cells and 30 ml medium per flask in vesicle-depleted culture medium after 4 h of culturing at 37 °C.

### MV analysis by TRPS

The concentration and size distribution of the MVs were determined by TRPS using the qNano Gold (Izon Science Ltd., Oxford, UK). This technique identifies individual particles in suspension by detecting a change in the voltage applied across a nanopore within a membrane when particle pass through this pore. Measurements were conducted using an NP150-rated pore with a fixed stretch of 47 mm, a transmembrane voltage of 0.48 V (led to a baseline current of ±100 nA), and a pressure of 6 mbar. To prevent particle aggregation, solution G (Izon Science Ltd. Oxford, UK) was added (10%) to the supernatants diluted (1:1) in solution Q (Izon Science Ltd. Oxford, UK), each sample was measured for 10 min. The samples were calibrated using 114 nm polystyrene calibration beads (CPC100, Izon Science Ltd. Oxford, UK) at a concentration of 1 × 10^9^ particles ml^-1^ diluted in culture medium. All reagents were purchased from Izon (Reagent kit (type RK1) for EV analysis, Izon Science Ltd., Oxford, UK). Data were analysed using Izon Control Suite Software v3.2 and concentration calculations were performed using Graph-Pad Prism 5 Software (Graph-Pad, San Diego, CA, USA) and Microsoft Office Excel (version 2010, Microsoft) and corrected for residual vesicles found to be present in the vesicle-depleted culture medium.

### Flow cytometric analysis of MVs using antibody-coated latex beads

A bead-based flow cytometric assay was used for the semi-quantitative analysis of CD63/CD81^+^ host cell-derived MVs. This method is based on the assay described previously [19, 20]. Briefly, 4-μm-sized aldehyde-sulfate beads were washed in MES buffer and coated with an antibody against CD63, a marker for host-cell MVs. Antibody-coated beads were incubated overnight with 200 μl of processed supernatant obtained after macrophage infection/stimulation, or with vesicle-free control medium. The supernatants were processed by centrifugation at 300×*g*, at 1200×*g,* and 0.22-μM filtration. During overnight incubation, the samples were kept under constant agitation at 6500 rpm at room temperature (RT). The next day, beads were washed twice with 0.22-μM filtered PBS with 2% (*w*/*v*) bovine serum albumin (BSA) and incubated under continuous shaking with PE-conjugated anti-CD81 (Abcam, Cambridge, MA, USA). Then, the beads were washed and diluted in 300 μl PBS for analysis by flow cytometry on a FACSCanto™ (BD Biosciences, Franklin Lakes, NJ, USA). Analyses were performed using FACSDiva Software and the threshold for the percentage of PE-positive beads was based on control beads incubated in culture medium and set at 2%. The relative amount of MVs, expressed in arbitrary units (AU), was calculated by the multiplication of the number of positive beads with the median fluorescence intensity.

### Pro-inflammatory and immuno-modulating characteristics

THP-1 macrophages were seeded in a 96-well plate, washed twice with PBS and provided with fresh complete vesicle-depleted culture medium with 5% FCS. Hereupon, cells were exposed to 20 μl of the SEC purified MVs (isolated after macrophage infection), bacterial MVs (bMVs), host-cell derived extracellular vesicles (EVs: isolated after macrophage stimulation with heat-inactivated bacteria) or whole heat-inactivated bacteria for 16 h. To determine the effect of MV pre-exposure on the response to a subsequent TLR-4 or TLR2/1 challenge with LPS or Pam3CSK4, respectively, THP-1 macrophages were cultured, treated, and stimulated as described above for a period of 4 h. After 4 h of stimulation with MVs, the cells were washed 3 times with PBS and after the addition of vesicle-depleted culture medium, re-stimulated with either LPS (100 ng ml^-1^) or Pam3CSK4 (50 ng ml^-1^) for 16 h. The vesicle amounts (20 μl) used for the THP-1 macrophage exposures represent 2 times the physiological concentration that is theoretically present in a physiological situation (i.e. the amount of vesicles that is present after an 4 h infection of 1 × 10^7^ THP-1 macrophages with either one of the bacteria at an MOI of 10). This volume represents this amount of vesicles is based on the recovery as determined by TRPS-based analysis of the vesicle concentration in conditioned culture medium and of the purified vesicles as obtained on vesicle enrichment using ultrafiltration and vesicle purification by SEC. The recovery was determined using MVs from infection experiments (Fig. 5d: MVs-SEC; based on NTHi and Mrc) and calibration beads (Fig. 5d: beads-SEC; based on 114 nm-sized Izon® calibration beads). The culture supernatants were harvested hereafter and used for cytokine measurements.

### The effect of vesicle exposure on the number of adherent and intracellular bacteria

THP-1 macrophages seeded in 24-well plates were washed 3 times with PBS after which 300 μl complete vesicle-depleted culture medium with 5% FCS was added. Then 200 μl of the MV-containing SEC fraction was added to the appropriate wells and the cells were pre-exposed to the vesicles for 1 h. Upon pre-exposure, the cells were infected with either one of the bacteria at a MOI of 50 for 1 h. After infection, the cells were washed 3 times with PBS to remove free bacteria, then cells were either left untreated, to determine the total amount of adherent and intracellular bacteria, or the cells were treated with gentamicin as described above to determine the number of intracellular bacteria. After bacterial plating of 2 different dilutions, the number intracellular bacteria was determined and the number of adherent bacteria was determined by subtracting the number of bacteria left after gentamicin treatment from the number obtained in the absence of gentamicin.

### Cytokine measurements

The release of TNF-α, IL-8, and IL-1β by THP-1 macrophages was determined by enzyme-linked immuno-sorbent assay (ELISA). The human Ready-Set-Go ELISA kits were obtained from eBiosciences and the assays were performed according the manufacturers’ instructions (Affymetrix eBioscience, Santa Clara, CA, USA).

### Cryo-electron microscopy

Upon MV isolation and purification by ultrafiltration and SEC the MV-containing SEC fractions were further concentrated to 100 μl using Amicon ultra-4 10-kDa centrifugal filter units (Millipore, Billerica, MA, USA). Vitrified specimens were prepared by loading a grid into an FEI Mark IV Vitrobot (FEI Company, Eindhoven, The Netherlands), adding 5 μl of MV-suspension to the grid, and then immediately blotting the grid for 1 s before plunge-freezing in liquid ethane which was kept at its melting point by liquid nitrogen using a Vitrobot environmental chamber that was maintained at 95% humidity. For microscopy, grids were mounted in a Gatan cryoholder in liquid nitrogen and transferred to a Tecnai T12 Spirit microscope (FEI Company, Eindhoven, the Netherlands). Images were acquired with a 4096 × 4096 pixel CCD Eagle camera (FEI Company, Eindhoven, the Netherlands) at 120 kV with a temperature between -170 °C and -175 °C. Image analysis to determine the median MV-diameter was performed using ImageJ processing software (National institutes of Health, Bethesda, USA).

### Statistical analysis

Statistical analysis was performed using Graph-Pad Prism 5 Software (Graph-Pad, San Diego, CA, USA). Statistical dispersion was determined by calculation of the standard error of the mean. One-way analysis of variance (ANOVA) was performed in combination with the Bonferroni multiple comparison test to determine statistical significance of the variances between multiple groups. An unpaired t-test was performed for the statistical analysis of the variance between the means of 2 groups. *P*-values were considered significant when <0.05.

## Results

**Fig. 1:**
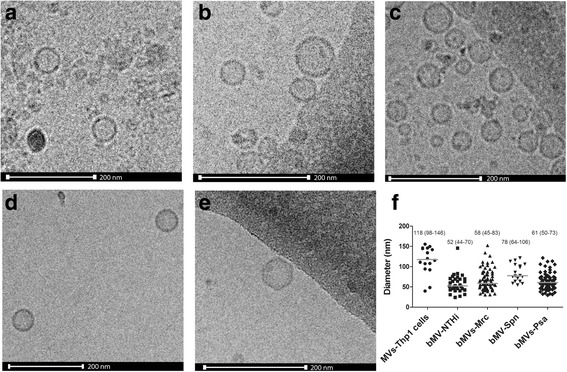
Cryo-EM analysis of MVs released by THP-1 macrophages (**a**) or during bacterial culture of NTHi (**b**), Mrc (**c**), Spn (**d**), and Psa (**e**). MVs released during bacterial culture show a similar size and appearance. The median is based on the diameter as obtained by cryo-EM imaging (**f**). Scale bar represents 200 nm

To study vesicle release during short-term infection of THP-1 macrophages and during culture of NTHi, Mrc, Spn, and Psa, we first aimed to characterize MVs shed by unstimulated THP-1 macrophages and during culture. Cryo-EM analysis revealed that THP-1 macrophages release MVs under control conditions and that the release of MVs occurs by all bacteria during culture (Fig. 1a-e). Also, the morphology of the bacterial MVs was highly comparable between the different species with an overall median diameter of 60 nm with an interquartile range (IQR) of 54–70 nm whereas the size of the THP-1 macrophages-derived MVs was found to be larger at 117 with an IQR of 98–146 nm (Fig. 1f).

**Fig. 2:**
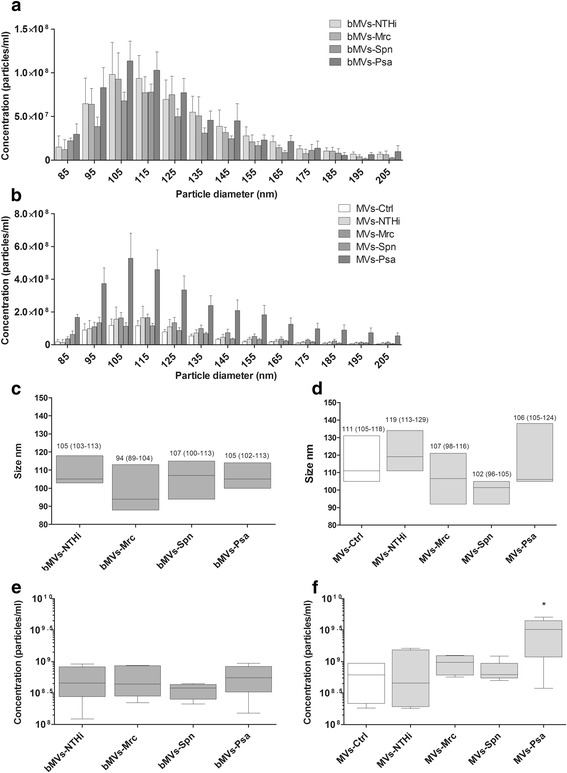
Tunable resistive pulse sensing-based characterization of MVs released upon infection with or culture of NTHi, Mrc, Spn, and Psa. Conditioned media of bacterial cultures (bMVs) were used to assess the vesicle size and numbers of vesicles released (**a**). Also, MV release by uninfected THP1 macrophages (MVs-Ctrl) and by THP1 macrophages after 4 h of infection with different bacteria (MVs-NTHi, -Mrc, -Psa, -Spn) was determined (**b**). The median diameter and interquartile range (between brackets) was calculated and is given for bacterial MVs (**c**) and for the mixed MV population released by cells and bacteria during infection (**d**). Box and whisker plots (**e**-**b**) indicate the median (line in box), 25th and 75th percentiles (outer lines of box) and the minimal and maximal values (whiskers) for the bacterial MV concentration (**e**) and the concentration of MVs released upon infection (**f**). Results are from 6 independent experiments. *p < 0.05

Next, we determined the size distribution and median size of MVs shed by unstimulated THP-1 macrophages and bacteria during 4 h of culture by TRPS. A similar distribution and size range was found for all bacterial MVs (NTHi: 105 (IQR 103-113), Mrc: 94 (IQR 89-104), Spn 107 (IQR 100-113), and Psa: 105 (IQR 102-113) nm) (Fig. 2a and c). Regarding the concentrations, although there were less MVs released by Spn (3.8 (IQR 2.5-4.3) ×10^8^ particles ml^-1^) as compared to the other bacteria (4.5 (IQR 2.8-8.2) ×10^8^ for NTHi, 4.4 (IQR 2.8-8.6) ×10^8^ for Mrc, and 5.5 (IQR 3.3-8.4) ×10^8^ particles ml^-1^ for Psa), this difference was not statistically significant (Fig. 2e).

Having characterized the MV release by both THP-1 macrophages and bacteria under control culture conditions, we continued with the characterization of the mixed MV populations released during infection. Macrophages were infected with the bacteria for 4 h after which the size and concentrations of the MV populations were determined. The size-distribution of the mixed MV populations shed during infection were found to be highly similar (Fig. 2b). Overall there was little variation in the MV size and the median size was found to be 107 nm with an IQR of 105-115 (Fig. 2d). With respect to the concentration, the absolute numbers tended to be somewhat increased upon infection with NTHi and Mrc compared to the aggregate number that was released by individual bacteria and THP-1 macrophages. This increase was, however, not significant (NTHi: 4.5 (IQR 1.9-15.3) ×10^8^, Mrc: 9.8 (IQR 6.1-12.4) ×10^8^ vs. uninfected: 6.2 (IQR 2.2-9.4) ×10^8^ particles ml^-1^) (Fig. 2f). Infection with Spn did not result in a change in the concentration of MVs (6.2 (IQR 5.5-9.4) ×10^8^ particles ml^-1^). THP-1 macrophage infection with Psa, on the other hand, significantly increased the MV release when compared to control conditions (Psa: 3.2 (IQR 1.2-4.5) ×10^9^ vs. uninfected: 6.2 (IQR 2.2-9.4) ×10^8^ particles ml^-1^, p≤0.05) (Fig. 2f).

**Fig. 3:**
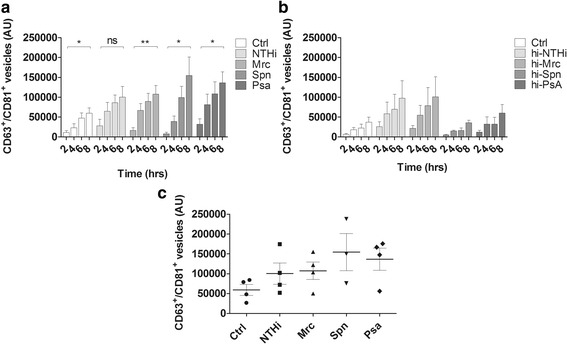
Time dependent increase in the release of CD63/CD81^+^ host cell MVs, by uninfected THP-1 macrophages (Ctrl), and by THP-1 macrophages upon exposure to viable or heat-inactivated NTHi, Mrc, Spn, and Psa. THP-1 macrophages were infected (**a**) or stimulated with (heat-inactivated) bacteria (**b**) at an MOI of 10 for 2, 4, 6, and 8 h. The supernatants were collected and used for bead-based flow cytometric analysis to determine (**a**) the relative number of CD63^+^/CD81^+^ MVs after infection (*n* = 4 except for spn (*n* = 3) or (**b**) stimulation with heat-inactivated bacteria (*n* = 3). After infection, the MV release increased over time and at all the 8-h time points the MV numbers for all conditions were significantly increased over the 2 h time points, except for NTHi. After stimulation with heat-inactivated bacteria, no significant increase was found between MVs release at 8 h and 2 h of stimulation (**c**). After 8 h of infection there was a tendency towards an increased release of MVs for all pathogens as compared to control, which was significant after Psa-infection (*n* = 4 except for spn: *n* = 3). Data are means ± the SEM. *p < 0.05, **p < 0.01

**Fig. 4:**
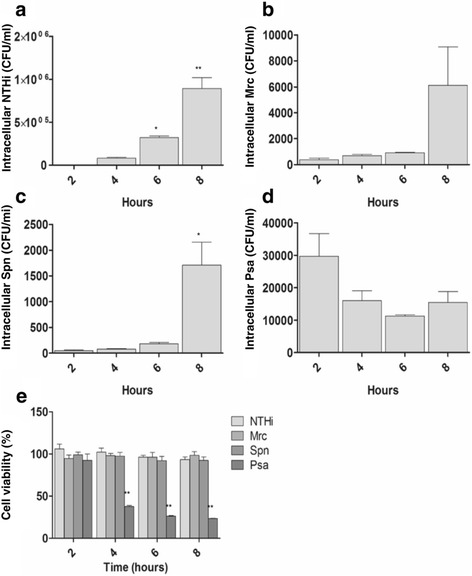
The number of intracellular bacteria and the cell viability over time after infection with various bacteria. THP-1 macrophages were infected for 2, 4, 6, and 8 h with NTHi, Mrc, Spn or Psa, then washed, treated with 300 μg gentamicin and lysed to determine the number of intracellular bacteria (**a**). The viability of THP-1 macrophages was determined over the course of infection (**b**). Data are represented as the mean ± SEM (*n* = 3). *p < 0.05, **p < 0.01

After the analysis of the whole MV population released during infection, the release of host-cell MVs over time, during infection, was determined. Using a semi-quantitative flow cytometry-based assay, we established the release of CD63^+^/CD81^+^ host-cell MVs: CD63 and CD81 are both members of the tetraspanin family and highly enriched on host cell-derived MVs. We found that the release of CD63^+^/CD81^+^ host cell MVs under control conditions was time-dependent and highest after 8 h (Fig. 3a). Likewise, during infection the release of vesicles also increased in a time-dependent way. For all conditions the vesicle release at 8 h was found to be significantly increased over the vesicle release at 2 h (except for the NTHi-based condition) (Fig. 3a). When compared to the release under control conditions, after 8 h of infection there was a tendency towards an increased host cell MV release by infected cells (Fig. 3c). Moreover, to determine whether viable bacteria were required for this induction, the MV release by THP-1 macrophages was determined following stimulation with heat-inactivated bacteria (Fig. 3b). Interestingly, although the number of MVs released by host cells following exposure to heat-inactivated NTHi or Mrc was not different from the number of MVs released following infection with viable bacteria, numbers were lower for THP-1 macrophages exposed to heat-inactivated Psa or Spn. These data suggest that, at least for Psa and Spn, viable bacteria are required to evoke an increased MV release by host cells. Finally, we determined the effect of infection on the cell viability and number of intracellular bacteria over the course of infection. We found that the cell viability was not altered by an infection with NTHi, Mrc, and Spn (Fig. 4e). Infection with Psa, however, led to a 60% reduction in cell viability after 4 h and a 70% reduction after 8 h of infection. Moreover, the number of intracellular bacteria was found to increase over time for all bacteria except for Psa. Recently it was shown that intracellular bacteria also release MVs [13], therefore these findings implicate that during infection, bacterial MVs can be released both by intra- and extracellular bacteria (Fig. 4a-d). Taken together our results show that both bacterial and host cell MVs are released during infection.

**Fig. 5:**
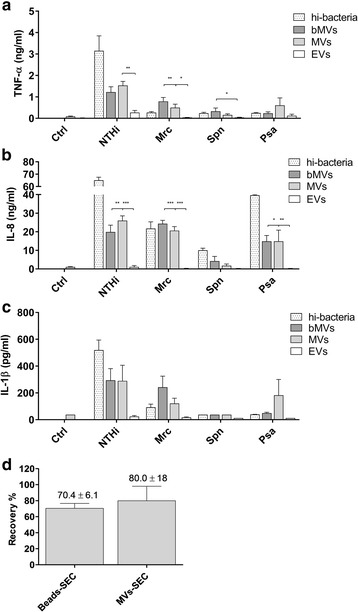
THP-1 macrophages were exposed for 16 h to MVs released during bacterial culture (bMVs), infection (MVs), vesicles from THP-1 macrophages that were stimulated with heat-inactivated bacteria (EVs), and to whole heat-inactivated bacteria at an MOI of 0.1 (hi-bacteria). Then the release of the pro-inflammatory cyto- or chemokines TNF-α (**a**), IL-8 (**b**) and IL-1β (**c**) was determined by ELISA. The number of MVs used for these exposures represents 2-times the physiological MV-concentration cells are theoretically exposed to when cells are infected with the bacteria at an MOI of 10. To calculate the amount of MVs to be used for these experiments we determined the vesicle recovery following vesicle-enrichment using ultrafiltration and vesicle purification by SEC using TRPS on MVs from infection experiments (MVs-SEC; based on NTHi and Mrc) and calibration beads (beads-SEC; based on 114 nm-sized Izon® calibration beads) (**d**). Data are represented as the mean ± SEM (*n* ≥ 4). *p < 0.05, **p < 0.01

Careful characterizations of the MV protein content in previous studies identified immunogenic bacterial proteins on MVs. Therefore, functional aspects of bacterial MVs, MVs derived from infected THP-1 macrophages and of MVs from THP-1 macrophages stimulated with heat-inactivated bacteria were studied. To this end, the release of several pro-inflammatory cyto- and chemokines by naive THP-1 macrophages upon MV exposure was assessed. The number of MVs used for these exposures represents 2-times the physiological MV-concentration that cells are theoretically exposed to when cells are infected with the bacteria at an MOI of 10 (as described in the materials and methods section). This amount is based on the vesicle recovery after SEC (Fig. 5d). Bacterial MVs elicited the release of TNF-α, IL-8 and IL-1β by naive THP-1 macrophages, albeit with different potencies for the different bacterial species (Fig. 5a-c, respectively). For example, stimulation with MVs derived from NTHi resulted in the release of >1 ng ml^-1^ TNF-α, while the amount of TNF-α release upon stimulation by Mrc and Psa MVs was far less pronounced and Spn MVs were even unable to induce a significant release of TNF-α. These variations were comparable with the variations observed on stimulation with whole (heat-inactivated) bacteria (Fig. 5a). When naive THP-1 macrophages were stimulated with MVs obtained after infection, the amounts of TNF-α released were almost identical to the amounts released when stimulated with bacterial MVs only. This suggests that the bacterial MVs are primarily responsible for activation of naive THP-1 macrophages, which is supported by the almost complete absence of TNF-α release following stimulation with MVs of THP-1 macrophages that were exposed to heat-inactivated bacteria, a vesicle population that exclusively consists of host cell-derived MVs.

**Fig. 6:**
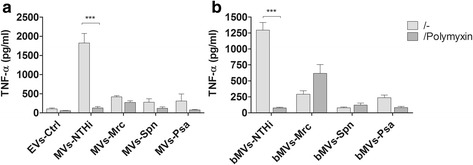
Effect of polymyxin B treatment on the TNF-α release in response to MVs released during infection or bacterial culture. THP-1 macrophages were pre-incubated for 1 h with polymyxin B prior to overnight stimulation with MVs released upon infection (MVs: **a**) or bacterial culture (bMVs: **b**). Data are represented as the mean ± SEM (*n* = 3). ***p < 0.001

Earlier it has been shown that several of the PAMPs associated with MVs are well-known ligands for Toll-like receptors (TLRs) (as reviewed by Ellis et al. [21]). Therefore, the next aim was to determine whether MV-associated LPS is responsible for the MV-induced TNF-α response. To neutralize the effects of MV-associated LPS, polymyxin B (a compound that binds to the lipid A region of LPS) was used. The specificity of polymyxin B was confirmed as it only blunted the LPS-induced TNF-α release by naive THP-1 macrophages (2.2 ± 0.3 to 0.2 ± 0.2 ng ml^-1^), but not the TNF-α release induced by the TLR2/1 ligand PAM3CSK4 (2.3 ± 0.1 to 1.9 ± 0.2 ng ml^-1^). Polymyxin B was found to significantly reduce the TNF-α release in response to NTHi MVs or MVs isolated from NTHi-infected THP-1 macrophages (Fig. 6a and b). The response to MVs from the Gram-positive Spn were not considerably affected. Moreover, the Mrc-MV-induced TNF-α response also remained unaffected.

**Fig. 7:**
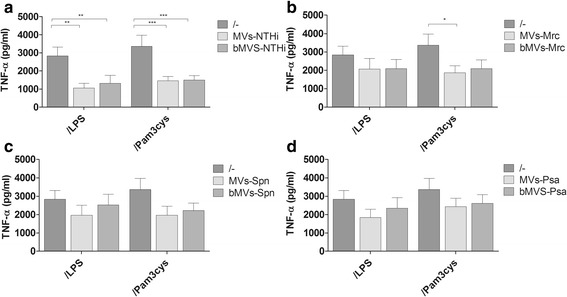
The pro-inflammatory response to a secondary challenge with LPS or Pam3CSK4 is blunted after THP-1 macrophages pre-exposure to MVs released during infection or bacterial culture. The TNF-α response to LPS or Pam3CSK4 upon MVs pretreatment was determined and is shown separately for bacterial MVs (bMVs) or mixed MVs isolated from THP-1 macrophages infected with NTHi (**a**), Mrc (**b**), Psa (**c**), and Spn (**d**). THP-1 macrophages were pre-incubated with purified MVs/bMVs for 1 h before secondary stimuli were added and the cells were exposed overnight to these components or to a combination. Data are represented as the mean ± SEM (*n* ≥ 4). *p < 0.05, **p < 0.01

To determine how bacterial MVs or MVs shed upon infection modulate a subsequent pro-inflammatory response to bacteria, THP-1 macrophages were pre-incubated with MVs for 4 h and subsequently exposed to LPS or Pam3CSK4, thereby mimicking bacterial infection. Pre-incubation with NTHi-derived MVs as well as MVs derived from NTHi-infected THP-1 macrophages significantly blunted the TNF-α response to both LPS and Pam3CSK4 (Fig. 7a). Moreover, pre-exposure to Mrc MVs also significantly reduced the response to secondary stimulation with Pam3CSK4 (Fig. 7b). Psa and Spn MVs or MVs from infected THP-1 macrophages did not significantly reduce the release of TNF-α in response to LPS or Pam3CSK4 (Fig. 7c-d, respectively). These results indicate that the presence of MV-associated TLR-agonists determines the subsequent response to a LPS or Pam3CSK4 challenge.

**Fig. 8:**
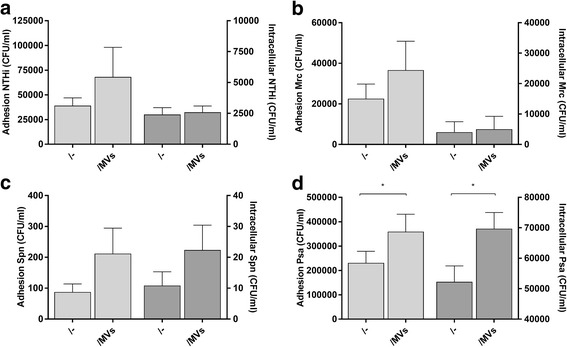
Number of adherent and intracellular bacteria after exposure of THP-1 macrophages to MVs released by infected THP-1 macrophages or bacteria during culture. The effect of MV-exposure on the adhesion and intracellular bacteria are shown separately for the conditions based on NTHi (**a**), Mrc (**b**), Spn (**c**), and Psa (**d**). THP-1 macrophages were pre-incubated with purified MVs/bMVs for 1 h before the cells were infected with either one of the bacteria (MOI 50) for 1 h in the presence of the vesicles. Bacterial counts (CFU/ml) were determined by plating of bacterial serial dilutions. Data are represented as the mean ± SEM (*n* ≥ 4). *p < 0.05

To further elucidate how MVs shed upon infection affect the course of infection, the next aim was to determine whether MV exposure affects the bacterial adhesion and the number of intracellular bacteria. To assess this, THP-1 macrophages were pre-exposed for 1 h to the MVs after which the bacteria were added. After another hour, the cells were washed and left either untreated (to assess for the number of adherent and intracellular bacteria) or were treated with gentamicin (to determine the number of intracellular bacteria). When exposed to MVs from NTHi, Mrc, and Spn there was a trend towards an increased bacterial adhesion but the intracellular bacterial load was not affected (Fig. 8a-c). However, exposure to MVs from Psa-infected cells significantly enhanced number of adherent and intracellular bacteria (Fig. 8d).

## Discussion

In this study, we demonstrated that a variety of bacterial species release MVs both during culture and infection of host cells. In addition, we show that THP-1 macrophages also release MVs, both under control conditions and during infection. Moreover, we showed that MVs released during infection have immuno-stimulating and -modulating properties.

Previous studies have shown that the shedding of bacterial MVs may provide bacteria with a selective advantage as it may aid bacterial invasion of host tissues (as reviewed by Kaparakis-Liaskos, 2015 [3]). In addition, during infection these vesicles can also evoke the release of pro-inflammatory cytokines as they carry PAMPs. MVs from *M. catarrhalis*, non-typeable *H. influenzae*, and *P. aeruginosa* have been detected in patients with various airway conditions suggesting that these MVs may also contribute to the pathogenesis of airway diseases [12, 22–24]. Regarding the dynamics and characteristics of bacterial MVs released upon long-term bacterial culture, these have been studied extensively [22, 25–28]. However, these characteristics are still poorly defined for MVs shed upon short-term infection. Moreover, MVs are also being released by the host both under control conditions and during infection, and it is unknown whether these MVs also exhibit immuno-stimulatory or -modulatory properties. Therefore, in this study we determined the release and functional properties of both bacterial and host cell MVs released upon infection of THP-1 macrophages with different bacterial species.

Analyses with both cryo-EM and TRPS confirmed the shedding of MVs upon bacterial culture and by THP-1 macrophages under control conditions. Strikingly, the median diameter of the bacterial MVs which was obtained by cryo-EM was found to be smaller than that obtained by TRPS (56.7 (IQR 53.7-73.4) vs 105.0 (IQR 95.5- 110.8) nm). It is possible that this discrepancy is introduced by the cutoff value of the lower threshold of the qNano (previously established at 70-100 nm [29]). This may have led to the inability of the qNano to discriminate signals generated by smaller MVs from the background noise, resulting in a size that was skewed towards a larger MV diameter. Regarding the total number of MVs released, TRPS analysis revealed a significant increase in the number of MVs released during Psa infection, and a similar tendency was observed for the other bacteria. Likewise, bead-based flow cytometric analysis also revealed that the release of CD63/CD81^+^ host cell MVs also increased upon infection. Remarkably, the magnitude of the Psa-induced increase release of CD63/CD81^+^ MVs was in the same range of the other conditions which does not explain the results obtained by TRPS analysis that showed an almost 10-fold increase upon Psa infection. In this study we also determined two other aspects that consider the macrophage infections namely the number of intracellular bacteria and the host-cell viability after infection. This latter revealed a high percentage of cell death already upon 4 h of Psa-infection, therefore it is likely that these vesicles are apoptotic bodies or nanosized membrane structures released from death or dying cells [30]. However, further research will be required to establish the origin of this MV population. This difference in cytotoxicity between Psa and the other bacteria could have been caused by differences in secretion system-associated virulence factors [31]. It has been shown that the cytotoxicity of Psa correlates with the expression of the type III secretion system which enables Psa to inject highly cytotoxic exotoxins into the host cell [32]. Moreover, we observed an increase in the number of intracellular bacteria (total number on bacterial invasion/phagocytosis, intracellular growth as well as bacterial killing after phagocytosis) over the course of infection for all bacteria except for Psa which likely resulted from its cytotoxicity. As it has been shown that the release of membrane vesicles by bacteria can also occur by intracellular bacteria, the bMVs released during macrophage infection could, therefore, have been originated from extra- as well as intracellular bacteria.

Another interesting observation made by TRPS-analysis is that there are no significant differences in the numbers of bacterial MVs shed by either the three Gram-negative bacteria or the Gram-positive Spn. This comparable release by both Gram-positive and Gram-negative bacteria might be an important finding as the release of MVs by Gram-positive bacteria has only been recognized recently (as reviewed by Brown et al. [8]). The release of MVs by Gram-negative bacteria is acknowledged as an evolutionarily conserved process for which at least 3 different mechanisms of release have been described. Future research needs to reveal whether the release of MVs by Gram-positive bacteria is likewise evolutionary conserved.

Earlier studies have demonstrated that next to external stressors (e.g. nutrient depletion) the biogenesis of MVs can be affected by the growth stage [22, 27, 28, 33, 34]. Most studies on MV shedding use bacterial cultures that are in the late exponential or stationary growth phase for the isolation of outer membrane vesicles [21, 26, 27, 35]. External stressors may also induce a growth stage transition towards stationary growth [36]. Our study shows that bacterial MV production also occurs during the early growth phase, both by the Gram-negative bacteria as well as by the Gram-positive Spn. Future research on the MV biogenesis and on factors that affect the MV release such as the growth phase and environmental factors will result in an improved understanding of the MV production and functional activity. To conclude, by using electron microscopic analysis, TRPS-based analysis, and flow cytometry we demonstrated that both bacterial and host cell MVs are released during infection.

The functional properties of bacterial MVs, including those released by the airway pathogens used in this study, have been studied previously (as reviewed by Ellis and Kuehn [21]). Moreover, studies have demonstrated the release of immuno-stimulatory host cell MVs during infection. These immuno-stimulatory properties of host cell-derived MVs have been attributed to the presence of several bacterial outer membrane proteins assumed to be present in these host cell vesicles [37–39]. Yet, this has recently been disputed by Athman and colleagues who demonstrated that host cells infected with *Mycobacterium tuberculosis* released two distinct MV populations, containing either host cell molecules (CD63/CD9) or bacterial components [13]. Here we determined the biological properties of the MVs shed upon infection and compared them to those of pure bacterial MVs shed during culture. Additionally, the immuno-stimulatory properties of MVs isolated from THP-1 macrophages following stimulation with heat-inactivated bacteria were determined. The MV population released by infected THP-1 macrophages elicited the release of significant amount of pro-inflammatory cytokines by naive macrophages, which was comparable to the amount of pro-inflammatory cytokine release following stimulation with pure bacterial MVs. We observed that this pro-inflammatory cytokine response was induced in a species-specific manner and that it was not induced by Spn-derived MVs. In line with previous studies we found that MVs released upon stimulation with heat-inactivated intracellular pathogens failed to induce a significant pro-inflammatory response [13, 40]. Overall, these results show that both host cell and bacterial MVs are released during infection and that particularly bacterial MVs have a strong immuno-stimulatory potential.

Studies that assessed the composition and function of bacterial MVs released upon culture identified several TLR-agonists on the membranes of these MVs [22, 26, 41–45]. Here, experiments with polymyxin B, a compound that antagonizes LPS, showed that the MV-induced pro-inflammatory response is predominantly dependent on the presence of LPS on MVs of NTHi and Psa but not Mrc. Our results are consistent with what has previously been published by Sharpe et al. who show that NTHi-derived MVs contain several immuno-stimulatory proteins including lipooligosaccharide (LOS) [35]. That LOS induces the release of TNF-α in a TLR-4 dependent manner was elegantly demonstrated in a study by Lorenz et al. [46]. In contrast, our results show that polymyxin B was not able to neutralize the Mrc-MVs in their ability to induce the release of TNF-α. This is in line with previous research that demonstrates the pro-inflammatory properties of these MVs are dependent on the presence of TLR-2 ligands [26]. Furthermore, previous studies on the composition and immunogenicity of Psa-derived MVs show that these vesicles can elicit potent pro-inflammatory responses due to the presence of several TLR-4 agonists [21], which supports our findings that polymyxin B reduced the TNF-α response to these MVs.

Previous studies that examined the effects of bacterial MVs demonstrate that a prolonged exposure to TLR-agonists-bearing MVs can also lead to TLR-desensitisation [47, 48]. In our study this was found to be pathogen dependent and mostly restricted to NTHi and Mrc. As a significant decrease was also observed upon pre-exposure to the analogous TLR-ligands, it is highly likely that this desensitisation was indeed TLR-mediated. A study by Waller et al. demonstrated that this TLR-2/4-induced desensitisation was dependent on IL-10 as the TNF-α responses was reinstated upon neutralization of IL-10. Moreover, it was shown that the IL-10 release induced on TLR-2 and TLR-4 activation, depended on a signalling pathway involving PI3K/PKB/Akt/mTOR [47]. We determined the levels of IL-10 following stimulation of naïve macrophages with a variety of host cell-derived and bacterial vesicles and only very low IL-10 levels (<5 pg/ml) were found under all conditions (results not shown). Therefore, we consider it unlikely that the observed MV-induced tolerance was IL-10 dependent*.*

Several studies investigating the functional properties of bacterial MVs demonstrated an enhanced bacterial adhesion upon MV exposure. The effects elicited by these MVs were shown to be mediated by vesicle-associated virulence [48, 49]. Likewise, we could demonstrate that MVs released by THP-1 macrophages during infection all tended to increase bacterial adhesion irrespective of the bacteria used. Moreover, the number of intracellular bacteria after exposure of THP-1 macrophages to MVs from Psa-infected cells was also found to be significantly increased. The latter requires a careful interpretation as on one hand it may indicate increase bacterial invasion while it also might be indicative for enhanced early phagocytosis. Further studies to examine the intracellular bacterial survival and determine the effects of our findings on the bacterial clearance will be required to establish the implications of our findings.

## Conclusion

In this study we determined the release and immunogenic properties of bacterial and host cell-derived MVs during short-term culture and infection. We demonstrated that bacterial MVs are released during bacterial culture and already within four hours of infection. Although both the mixed vesicle population released upon infection as well as pure bacterial MVs were found to have a profound pro-inflammatory potential, pre-exposure to these MVs led to a significant reduction in the pro-inflammatory response to a secondary challenge. Moreover, MVs released upon infection also led to an increased number of adherent and intracellular bacteria. Further research will be required to elucidate whether and how these MVs serve bacteria to overcome the host-immune response. A better understanding of these processes may help to find new therapies to combat bacterial respiratory infections.

## Abbreviations

AU: Arbitrary units
CFU: Colony forming units
EM: Electron microscopy
EVs: Extracellular vesicles
FCS: Fetal calf serum
IL-1β: Interleukin-1β
IL-8: Interleukin-8
IQR: Interquartile range
LOS: Lipooligosaccaride
LPS: Lipopolysaccharide
MOI: Multiplicity of infection
Mrc: *Moraxella catarrhalis*
MVs: Membrane vesicles
Nm: Nanometer
NTHi: Non-typeable *Haemophilus influenzae*
Psa: *Pseudomonas aeruginosa*
SEC: Size exclusion chromatography
Spn: *Streptococcus pneumoniae*
TLR: Toll-like receptor
TNF –α: Tumor necrosis factor-α
TRPS: Tunable resistive pulse sensing
